# The importance of social zeitgeber in paediatric type 1 narcolepsy: What we can learn from the COVID‐19 restrictions adopted in Italy?

**DOI:** 10.1111/jsr.13423

**Published:** 2021-06-22

**Authors:** Marco Filardi, Anita D'Anselmo, Alice Mazzoni, Monica Moresco, Fabio Pizza, Giuseppe Plazzi

**Affiliations:** ^1^ Department of Biomedical and Neuromotor Sciences (DIBINEM) University of Bologna Bologna Italy; ^2^ IRCCS Istituto delle Scienze Neurologiche Bologna Italy; ^3^ Department of Biomedical, Metabolic and Neural Sciences University of Modena and Reggio Emilia Modena Italy

**Keywords:** actigraphy, COVID‐19, lockdown, narcolepsy, sleep–wake schedules, social jetlag

## Abstract

The lockdown due to the new coronavirus pandemic (COVID‐19) has led to unparalleled changes in several aspects of human behaviour. During the lockdown, the general population delayed sleep timing and spent more time in bed; however, little is known on the effects of COVID‐19 restriction on children and adolescents suffering type 1 narcolepsy. In the last months of 2019, we performed follow‐up actigraphy in 18 type 1 narcolepsy children and adolescents under stable pharmacological treatment with sodium oxybate. We contacted these patients for a follow‐up actigraphy during the first Italian lockdown. Actigraphs and the Epworth Sleepiness Scale for children and adolescents (ESS‐CHAD) have been sent to participants’ homes. Differences in motor activity were analysed through functional linear modelling. During lockdown, type 1 narcolepsy children and adolescents went to bed and woke up later, slept more during the daytime and napped more frequently. No difference emerged in time in bed, estimated total sleep time and nocturnal sleep quality. Similarly, no difference emerged in ESS‐CHAD and body mass index. The time‐series analysis of motor activity documented reduced activity during the early morning and in the evening during the lockdown period compared with pre‐lockdown. Our study objectively showed that type 1 narcolepsy children and adolescents delayed the sleep phase and slept more during the daytime during the lockdown. The analysis of type 1 narcolepsy children and adolescents’ behaviour during the lockdown has provided new information that could pave the way to a personalized school programme.

## 1 INTRODUCTION

Type 1 narcolepsy (NT1) is a central disorder of hypersomnolence characterized by excessive daytime sleepiness, dissociated rapid eye movement (REM) sleep manifestations (cataplexy, sleep paralysis and hypnagogic/hypnopompic hallucinations) and disrupted nocturnal sleep (Scammell, 2015).

In a large percentage of cases, symptoms arise during childhood and early adolescence (Thorpy & Krieger, 2014). NT1 children and adolescents are frequently overweight or obese, and have difficulties in school and in integration with peers (Vignatelli et al., 2019).

In the last months of 2019, a new coronavirus disease (COVID‐19), firstly identified in China, quickly spread worldwide forcing several countries to impose national lockdown.

In Italy, the first and strictest lockdown took effect on 11 March and lasted till 18 May 2020: during this period, people were not allowed to leave their home except for limited and documented purposes. Educational activities, from primary school to university, were replaced with remote learning (Decreto del presidente del consiglio dei ministri, 2020).

The restrictions due to the COVID‐19 pandemic have led to unparalleled changes in several aspects of human behaviour. The effects of COVID‐19 restrictions on sleep have been assessed in several studies, often through retrospective questionnaires. During the lockdown, people delayed sleep phase and spent more time in bed (TIB; Blume, Schmidt, & Cajochen, 2020), but at the same time reported a worsening of sleep quality (Cellini, Canale, Mioni, & Costa, 2020). Suspecting that NT1 children and adolescents might be more susceptible to the COVID‐19 lockdown, also in light of the increased ultradian drive to sleep (Filardi, Pizza, Bruni, Natale, & Plazzi, 2016), we aimed to contribute to the knowledge on the impact of COVID‐19 restrictions on NT1 children and adolescents by objectively assessing nocturnal sleep, napping behaviour and motor activity pattern through actigraphy, a methodology that has already proved useful in providing information on disease course, and in monitoring effects and adherence to pharmacological treatments (Filardi, Pizza, Antelmi, Ferri, et al., 2018; Filardi, Pizza, Antelmi, Pillastrini, et al., 2018).

## 2 METHODS

Between November 2019 and January 2020, we performed routine follow‐up actigraphy in 30 NT1 children and adolescents (mean age: 13.27 ± 3.47 years) under stable pharmacological treatments with sodium oxybate. Data collection ended on 30 January, 24 days before the closure of schools (23 February) and the subsequent first national lockdown (11 March–18 May 2020). We therefore decided to follow up these patients during the lockdown period.

Eighteen NT1 children and adolescents (11 males, mean age: 14.44 ± 2.01 years, range 10–17 years) accepted to participate and to wear an actigraph continuously for 14 days. Actigraphs (Micro Motionlogger Watch, Ambulatory Monitoring) have been sent to participants’ homes, along with the Epworth Sleepiness Scale for children and adolescents (ESS‐CHAD) and the Italian version of the consensus sleep diary, which participants have to fill in daily (Carney et al., 2012; Wang, Benmedjahed, & Lambert, 2017). Moreover, participants were asked to report their height and weight upon receiving the device.

Actigraphic recordings have been processed with the Sadeh algorithm to obtain estimated sleep measures. We computed, separately for weekdays and weekend, bedtime, wake‐up time and midpoint of sleep (MS, the middle time‐point between bedtime and wake‐up time). Social jetlag was computed as the difference between MS of weekend and of weekdays (Wittmann, Dinich, Merrow, & Roenneberg, 2006).

We considered the following estimated nocturnal sleep measures: TIB, total sleep time (*e*TST), wake after sleep onset (*e*WASO), sleep efficiency, awakenings and prolonged (lasting more than 5 min) awakenings, longest uninterrupted sleep episode and sleep motor activity (SMA). For the diurnal part of the recording, we considered diurnal motor activity (DMA), estimated diurnal total sleep time (eDTST), estimated nap frequency, and mean nap duration (NapD).

The study was approved by the local health trust's ethics committee (Comitato Etico Interaziendale Bologna‐Imola, CE‐BI, Prot. Num. 17009), and written informed consent was obtained from the children's parents.

### 2.1 Statistical analysis

Continuous and categorical data were explored with descriptive statistics (mean ± SD). Differences between pre‐lockdown and lockdown period in ESS‐CHAD, body mass index (BMI) and actigraphic parameters were assessed by means of paired samples *t*‐test followed by effect size computation (Hedges’ *g*) in case of statistically significant results. To assess changes in motor activity profile, we processed the time‐series of raw motor activity data through functional linear modelling (FLM; Wang et al., 2011). Firstly, we extracted raw motor activity data through the Action‐4 software (Ambulatory Monitoring); to avoid variations related to the weekend, we considered solely data from Sunday midnight to Friday evening (corresponding to the regular school week in Italy). The five vectors of motor activity data were averaged into a single activity profile and fitted using a Fourier expansion model with *n* = 19 basis permutations. Differences in motor activity were assessed through non‐parametric permutation *F*‐test. Finally, we fitted a FLM on the motor activity profile of NT1 children and adolescents during the lockdown period according to differences in the ESS‐CHAD scores between the two evaluations (ᐃESS‐CHAD: ESS‐CHAD of the lockdown minus ESS‐CHAD of the pre‐lockdown). Statistical analyses were performed with R and SPSS 19.0 (SPSS); *p*‐values < .05 were considered statistically significant.

## 3 RESULTS

Actigraphic parameters, ESS‐CHAD score and BMI values are reported in Table 1.

**TABLE 1:** Actigraphic parameters, ESS‐CHAD score and BMI of NT1 children and adolescents prior and during the COVID‐19 lockdown

	Before lockdown (*n* = 18) Mean ± SD	During lockdown (*n* = 18) Mean ± SD	*p*	Hedges’ *g*
ESS‐CHAD	13.11 ± 3.18	12.94 ± 3.23	ns	
BMI	22.66 ± 4.73	22.60 ± 4.37	ns	
Sleep timing – weekdays				
Bedtime	23:06 ± 00:33	1:20 ± 00:43	< .0001	1.89
MS	2:56 ± 00:23	4:07 ± 00:37	< .0001	2.24
Wake‐up time	6:48 ± 00:29	7:45 ± 00:45	< .0001	1.70
Sleep timing – weekend				
Bedtime	23:46 ± 00:41	1:48 ± 1:03	< .005	1.12
MS	3:40 ± 00:38	4:41 ± 00:53	< .005	1.02
Wake‐up time	7:35 ± 00:54	8:34 ± 00:58	< .001	1.29
Social jetlag	43.08 ± 23.18	26.32 ± 17.25	< .05	−0.70
Nocturnal rest period				
TIB, min	462.14 ± 43.87	470.40 ± 51.95	ns	
*e*TST, min	375.04 ± 50.96	374.08 ± 54.38	ns	
*e*Sleep efficiency	81.29 ± 8.44	79.99 ± 10.53	ns	
*e*WASO, min	70.17 ± 37.48	80.47 ± 52.16	ns	
*e*Awakenings, n°	15.58 ± 7.92	15.61 ± 7.23	ns	
*e*Prolonged awakenings, n°	4.09 ± 2.21	4.44 ± 2.36	ns	
*e*Longest sleep, min	126.39 ± 34.87	133.47 ± 36.22	ns	
SMA, counts	23.32 ± 8.23	25.37 ± 11.35	ns	
Daytime period				
DMA	200.53 ± 23.78	173.61 ± 25.08	< .0001	−1.08
*e*DTST, min	59.81 ± 31.77	82.99 ± 49.13	< .05	0.55
*e*Nap, n°	2.89 ± 1.49	4.39 ± 2.73	.01	0.67
*e*Nap – weekdays	3.61 ± 1.85	5.89 ± 1.99	< .0005	−1.04
*e*Nap – weekend	1.88 ± 0.96	2.33 ± 0.76	ns	
*e*NapD, min	30.27 ± 12.32	34.92 ± 10.28	ns	

BMI, body mass index; DMA, mean activity counts during daytime; *e*DTST, estimated diurnal total sleep time; *e*NapD, mean *e*Nap duration; ESS‐CHAD, Epworth Sleepiness Scale for children and adolescents; *e*TST, estimated total sleep time; *e*WASO, estimated wake after sleep onset; MS, midpoint of sleep (the middle time‐point between bedtime and wake‐up time); SMA, mean activity counts during TIB; Social jetlag, MS of weekend minus MS of weekdays; TIB, time in bed.

None of the patients discontinued treatment with sodium oxybate during the lockdown, but we observed a slight increase in daily intake (7.25 ± 0.75 versus 7.42 ± 0.72, *t*_(17)_ = −2.06, *p* = ns). No statistically significant differences were observed in BMI (*t*_(17)_ = 0.30, *p* = ns) and ESS‐CHAD (*t*_(17)_ = 0.27, *p* = ns). Similarly, no differences were observed in actigraphic measures reflecting sleep quality (*e*TST, *e*WASO, estimated sleep efficiency, longest uninterrupted sleep episode, awakenings and prolonged awakenings). Significant differences emerged in sleep timing with NT1 children and adolescents who went to bed and woke up significantly later during the lockdown period on both weekdays and weekend (all *p* < .005). Social jetlag decreased during the lockdown period (*t*_(17)_ = 2.36, *p* < .05). Differences also emerged in actigraphic diurnal measures, with NT1 children and adolescents displaying lower DMA levels (*t*_(17)_ = 5.75, *p* < .0001), higher *e*DTST (*t*_(17)_ = −2.59, *p* < .05) and increased naps frequency (*t*_(17)_ = −2.89, *p* = .01), while mean nap duration was unchanged (*t*_(17)_ = −1.46, *p* = ns). The difference in nap frequency is due to a greater increase in naps on weekdays during the lockdown compared with the pre‐lockdown (*t*_(17)_ = −4.41, *p* < .0005).

**FIGURE 1:**
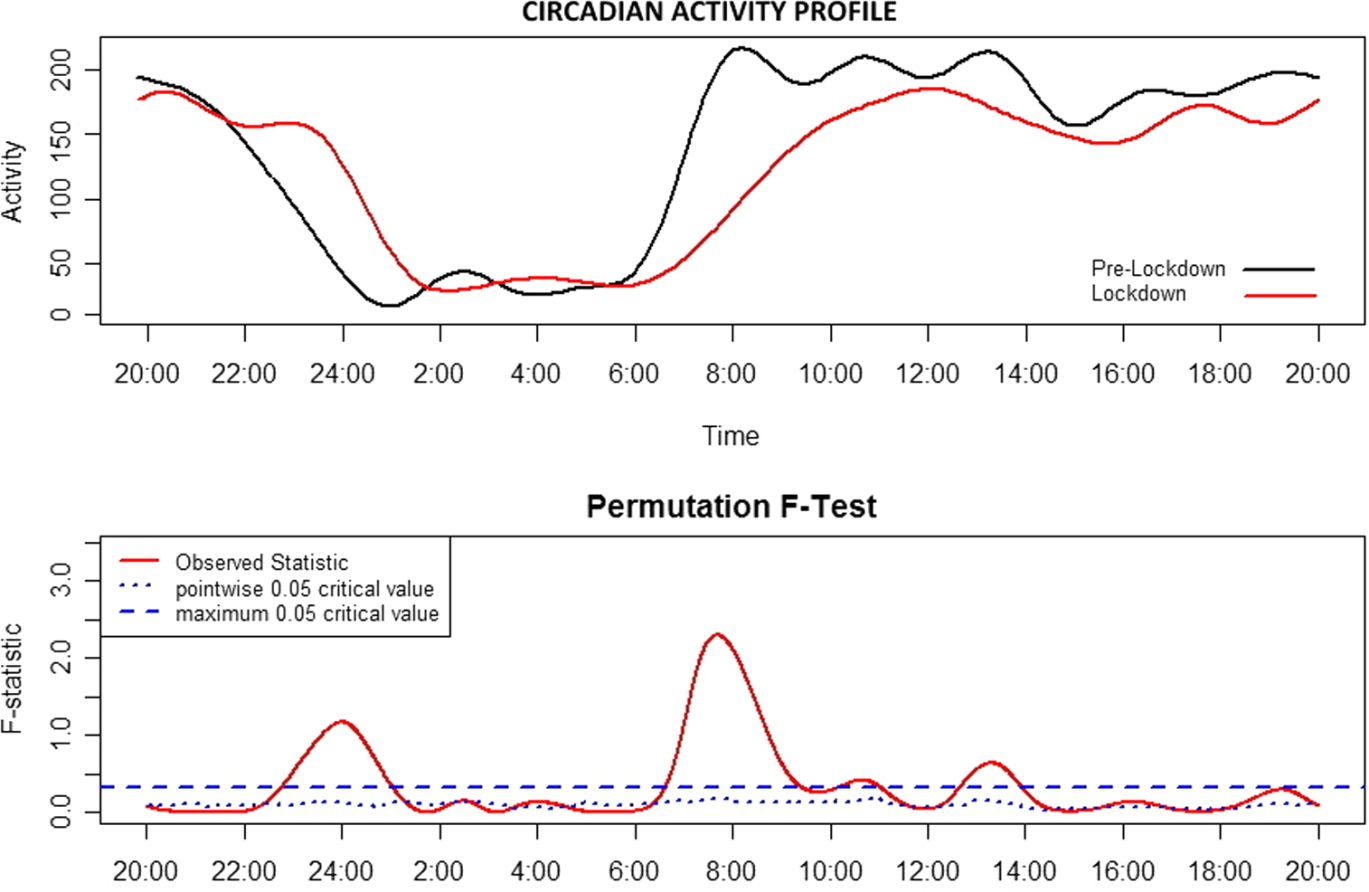
Circadian motor activity profile of type 1 narcolepsy (NT1) children and adolescents prior and during the lockdown. Lower panel: solid red line: observed statistic; blue dashed line: global test of significance; blue dotted line: point‐wise test of significance. Whenever the observed statistic is above the point‐wise or global threshold of significance, the activity profiles are significantly different at that specific time‐point

**FIGURE 2:**
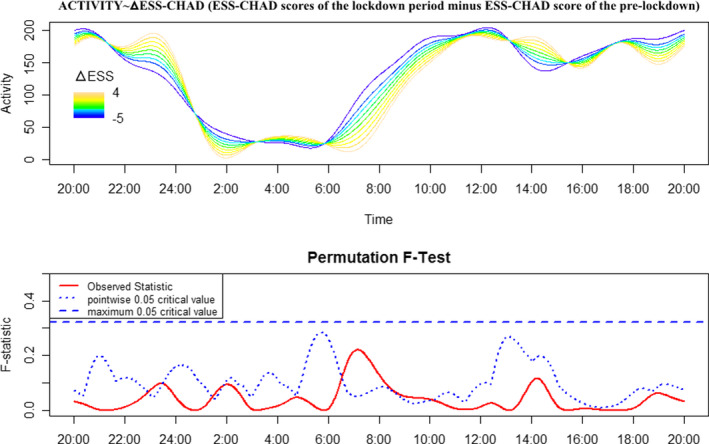
Motor activity profile of type 1 narcolepsy (NT1) children and adolescents during the lockdown period according to continuous ᐃESS‐CHAD values. Lower panel: solid red line: observed statistic; blue dashed line: global test of significance; blue dotted line: point‐wise test of significance. Whenever the observed statistic is above the point‐wise or global threshold of significance, a statistically significant association between motor activity and ᐃESS‐CHAD values is detected at that specific time‐point

The motor activity profile of NT1 children and adolescents prior and during the lockdown period is reported in Figure 1, together with the *F*‐test result. NT1 children and adolescents presented significantly higher motor activity from 22:30 hours to 01:45 hours, and lower motor activity levels from 06:30 hours to 09:45 hours (global test of significance) during the lockdown compared with pre‐lockdown. Moreover, NT1 children and adolescents displayed lower motor activity levels from 10:00 hours to 11:00 hours, 12:40 hours to 14:00 hours (global test of significance), and again from 18:30 hours to 20:00 hours (point‐wise test of significance) during the lockdown compared with pre‐lockdown. Finally, the FLM results for ᐃESS‐CHAD treated as a continuous variable are reported in Figure 2. A reduction of ESS‐CHAD is associated with higher motor activity from 06:15 hours to 10:00 hours (point‐wise test of significance).

## 4 DISCUSSION

Our study is the first to have objectively assessed the effects of COVID‐19 lockdown on nocturnal sleep, daytime napping and motor activity profile of NT1 children and adolescents.

Overall, our results are in line with those of questionnaire studies on adult NT1 patients and healthy subjects, in documenting a significant delay of sleep phase during the COVID‐19 lockdown (Blume et al., 2020; Rodrigues Aguilar et al., 2020). Indeed, NT1 children and adolescents delayed bedtimes by 75 min during weekdays and about 60 min during the weekend, while wake‐up times were delayed by about 60 min during both weekdays and weekends of the lockdown period. Social jetlag (i.e. differences between MS of weekdays and weekends) decreased by 17 ± 30 min.

However, despite the delayed sleep phase, NT1 children did not spend more TIB during the lockdown period. Similarly, we did not observe a difference in *e*TST and in actigraphic measures reflecting nocturnal sleep quality. This last result apparently contrasts with previous studies on adult NT1 patients and healthy controls, which reported increased sleep fragmentation during the COVID‐19 lockdown (Cellini et al., 2020; Rodrigues Aguilar et al., 2020). A possible explanation is that all patients included in this study were under stable pharmacological treatment with a powerful sedative‐hypnotics agent (sodium oxybate) that could have flattened any possible effect of COVID‐19 lockdown on sleep quality.

Likewise, we did not observe changes in subjective sleepiness between the pre‐lockdown and the lockdown period. To date, only two studies have assessed whether the COVID‐19 lockdown modifies sleepiness in NT1 patients, and reported conflicting results: the study by Postiglione et al. showed that adult NT1 patients who worked/studied at home during the lockdown period display lower subjective sleepiness compared with the pre‐lockdown (Postiglione et al., 2020), while the study by Rodrigues Aguilar et al. reported a worsening of subjective sleepiness during the lockdown period in a mixed sample of adult NT1 and NT2 patients (Rodrigues Aguilar et al., 2020).

Unique to our study design is the possibility to objectively study changes in diurnal sleep pattern and motor activity profile of young NT1 patients while confined at home.

Overall, NT1 children and adolescents slept more during the daytime and napped more frequently during the lockdown, but the duration of naps remained similar to the pre‐lockdown period.

On the other hand, mean motor activity levels decreased during the lockdown.

The time‐series analysis of motor activity (Figure 1) nicely depicts the changes that occurred in the behaviour of NT1 children and adolescents during the lockdown. Aside from the clear shift of sleep phase, NT1 children were less active during the first hours of the morning and in the evening between 18:00 hours and 20:00 hours. The result of the FLM model for ᐃESS‐CHAD scores treated as a continuous variable is reported in Figure 2: lower ᐃESS‐CHAD values are associated with higher motor activity in the early morning, in correspondence to online lesson times. Accordingly this result seems to indicate that school represents an important social zeitgeber for NT1 children and adolescents.

Noteworthy, no difference was observed in the hours corresponding to the postprandial period, when NT1 children slept independently of the changes in daily activities: at this time of the day NT1 children are quite fragile and vulnerable to sleep and therefore this should be considered with particular importance with a view to personalized school planning for NT1 children and adolescents. In addition to the above‐described results, our study suggests a number of recommendations that could prove useful in case of future lockdown periods. First, promote good sleep hygiene (i.e. regular sleep times, limited screen time in the evening), which will help NT1 children and adolescents to maintain a sleep–wake cycle synchronized during the lockdown.

Second, promote a regular schedule of at‐home physical exercise; albeit in our study the decrease in motor activity was not associated with an increase in BMI, probably also due to the proximity between observations; the promotion of regular physical exercise is of the utmost importance in a population that is prone to weight gain (Filardi, Pizza, Antelmi, Ferri, et al., 2018; Filardi, Pizza, Antelmi, Pillastrini, et al., 2018).

Our study also suffers several limitations. First, the sample size is relatively small, which makes problematic the comparison with previous studies (Postiglione et al., 2020; Rodrigues Aguilar et al., 2020). Second, we could not compare the severity of NT1 symptoms (apart from sleepiness) and children's quality of life, as these measures were not routinely collected in NT1 children and adolescents.

Similarly, we did not have collected data on NT1 children's eating habits, screen time and academic performances before and during the lockdown. The analysis of the behaviour of NT1 children and adolescents during lockdown has provided new information on the spontaneous sleep–wake organization of these children in the absence of social factors, and suggests that a personalized school‐time schedule might favourably impact on the academic performance of NT1 children and adolescents.

## CONFLICT OF INTEREST

Giuseppe Plazzi participated in the advisory board for UCB Pharma, Jazz Pharmaceuticals, Bioprojet and Idorsia outside the submitted work. The other authors have no potential financial conflict of interest to disclose.

## AUTHOR CONTRIBUTIONS

M.F.: conception of the study, statistical analysis, interpretation of data, drafting the initial manuscript and revising it for important intellectual content. A.D.: acquisition of data, interpretation of data, reviewed the manuscript for intellectual content. A.M.: acquisition of data, data curation, reviewed the manuscript for intellectual content. M.M.: data curation, interpretation of data, reviewed the manuscript for intellectual content. F.P.: data curation, interpretation of data, reviewed the manuscript for intellectual content. G.P.: conception of the study, study supervision, drafting the initial manuscript and revising it for important intellectual content.

## Data Availability

Data are available from the corresponding author upon reasonable request.
